# Efficient Sensor Placement Optimization Using Gradient Descent and Probabilistic Coverage

**DOI:** 10.3390/s140815525

**Published:** 2014-08-21

**Authors:** Vahab Akbarzadeh, Julien-Charles Lévesque, Christian Gagné, Marc Parizeau

**Affiliations:** Laboratoire de vision et systèmes numériques, Département de génie électrique et de génie informatique, Université Laval, Québec, QC G1V 0A6, Canada;E-Mails: vahab.akbarzadeh.1@ulaval.ca (V.A.); julien-charles.levesque.1@ulaval.ca (J.-C.L.); marc.parizeau@gel.ulaval.ca (M.P.)

**Keywords:** sensor placement, gradient descent optimization, line-of-sight coverage

## Abstract

We are proposing an adaptation of the gradient descent method to optimize the position and orientation of sensors for the sensor placement problem. The novelty of the proposed method lies in the combination of gradient descent optimization with a realistic model, which considers both the topography of the environment and a set of sensors with directional probabilistic sensing. The performance of this approach is compared with two other black box optimization methods over area coverage and processing time. Results show that our proposed method produces competitive results on smaller maps and superior results on larger maps, while requiring much less computation than the other optimization methods to which it has been compared.

## 1. Introduction

Recent years have seen a proliferation of interest in the use of sensor networks (SN) for different application areas, such as battlefield surveillance, factory automation and environmental monitoring, among others [1]. SNs consist of a network of sensor devices, where each device can autonomously sense the target environment and communicate with other sensors to achieve the goal of delivering valuable information to the end user.

Different issues need to be addressed when deploying a SN, such as localization, data fusion and placement. In the placement problem, the goal is to find the optimal position and orientation of the sensors in the target environment. Optimal placement of sensors is an important design issue, because it directly affects the operational performance of the SN, through the overall coverage of the network [2].

Coverage represents the performance of the network, because sensors are placed inside the environment to sense a phenomena, and coverage measures the quality of the service provided by the SN. There have been several definitions for coverage depending on the application [3,4], such as the area coverage, point coverage, barrier coverage, *k*-coverage and least exposure coverage. In area coverage, which is the most widely used criterion, the ratio between the area covered by the sensors to the total target area is used as the objective to be maximized. Point coverage and barrier coverage could be considered as special cases of the area coverage, in which special locations in the environment have higher importance in the coverage problem. Therefore, if one finds a general solution for the area coverage problem, it can be easily extended to point coverage and barrier coverage problems. In *k*-coverage, each location in the environment should be covered by at least k sensors. This is the requirement for some applications with high security (e.g., military surveillance applications), so that in the case of the failure of one sensor, the security of the area is not compromised. Finally, the goal in least exposure coverage is to find a path within the environment with low observability from sensor nodes. This problem is usually formulated as the worst-case coverage for a target that is moving between two points in the environment. In this paper, we use the area coverage as the performance criterion, because it is the most widely used performance measure, and other measures could be considered as special cases of area coverage (except *k*-coverage and least exposure coverage, which are designed for special applications). Hereinafter, the terms area coverage and performance are used interchangeably.

Determining the coverage of a SN also depends on the coverage model used for each sensor. The common assumption [5] is that each sensor can sense a circular area around itself having a radius known as the coverage range. This assumption of omnidirectional sensing ability does not hold true for many types of sensors, such as cameras, ultrasonic sensors, *etc.*, which have a directional sensing region. The other assumption is related to the detection ability of a sensor inside its sensing area. The conventional approach assumes a binary 0/1 coverage for each sensor [6], while a probabilistic coverage [7,8] better complies with the performance of real sensors in the environment.

The last assumption concerns the dimensionality of the target environment. The target area for the SN is a three-dimensional environment, and the simplification that sensors are placed in a two-dimensional environment [9] usually results in an overestimated performance of the network in real settings. In a realistic setting [10], the covered area of each sensor should also take into consideration the topography of the environment and obstacles occluding the sensing area of each sensor.

Optimal sensor placement has also been an active research area in civil infrastructure monitoring [11,12]. The sensors are placed in the structure to measure a wide range of properties, such as stress, displacement, acceleration, *etc.* The information gathered from the sensors is used within a continuous structural health monitoring system, which detects any structural damage before it becomes critical. In these applications, the coverage region of a sensor is not “local”. In other words, sensor readings from parts of the infrastructure far from each other might be highly correlated. Therefore, information theoretic criteria are usually used to evaluate the performance of a given placement. Some of the proposed criteria include the modal assurance criterion [13], information entropy [14] and the Fisher information matrix [15].

The sensor placement problem has also been addressed in the computational geometry field. Specifically, Voronoi diagrams and Delaunay triangulation have been used to estimate the coverage region of a sensor [16]. Considering the structure of Voronoi diagrams, each sensor is responsible to cover its Voronoi cell, because all of the points inside each cell are closer to the sensor generating that cell compared to other sensors. It can be easily concluded that if all Voronoi vertices of a Voronoi cell are within the coverage range of sensor, then there are no coverage holes inside that SN. Extending this idea, Wang *et al.* [17] proposed an effective heuristic to estimate the relative size of coverage holes using the distance between each sensor and its furthest Voronoi vertex. Using the same heuristic, a simple approach to heal the Voronoi holes is to move each sensor toward its farthest Voronoi vertex.

The placement problem in SNs is closely related to the observer sitting problem, which has been addressed in the geomatics science literature [18,19]. In this problem, one tries to find the optimal position for a number of observers, required to cover a certain ratio of an area. Methods proposed for this problem have been applied to determine the location of telecommunication base stations [20], to protect endangered species [21] and to determine the location of wind turbines [22]. Therefore, solutions proposed for the placement problem in SNs have great influence in related problems from other domains.

Methods proposed for placement optimization can be classified into two categories: exact methods and heuristic-based approaches. A group of approaches have considered the placement problem as a special case of the maximum coverage problem [23,24]. In these approaches, the problem is formulated so that a greedy algorithm can produce near-optimal results with approximation boundaries. Other algorithms, such as integer linear programming [25] and binary integer programming [26] methods, have also been employed for the placement problem. The shortcoming of these algorithms is that the assumptions about the environment and the sensors are very simplistic (e.g., two-dimensional target environments, binary coverage of sensors, *etc.*).

Another approach is based on the virtual potential fields [27]. In this strategy, sensors are moved by the repulsive forces that they sense from other sensors and the obstacles in the environment. These repulsive forces tend to spread sensors across the environment. At the same time, sensors sense a viscous friction force, which helps the sensors reach a static equilibrium.

A wide variety of meta-heuristic methods have also been applied to the placement problem, ranging from genetic algorithm [28], evolution strategies [10], evolution algorithm with specialized operators [29–31], swarm optimization [32,33] and simulated annealing [34]. An issue with meta-heuristic algorithms is their high computational cost, because these optimization algorithms usually need many evaluations of candidate solutions through simulations, which requires high processing resources. This issue makes these algorithms unsuitable for on-line applications where the position and orientation of sensors should be adapted after deployment and during utilization. The computational requirement also limits the size of the networks for which a solution can be found in a reasonable time.

In another set of approaches, spatial phenomena are modelled using Gaussian processes (GP). As a solution for the placement problem under this assumption, sensors could be placed in locations with highest entropy [35], or maximum mutual information [36]. Krause *et al.* [36] have shown a polynomial-time algorithm for the placement problem, which is within a constant factor of the optimal result. Even though the complexity of this algorithm is reduced to *O*(*kn*) for k sensors in n possible sensor locations (*n* ≫ *k*), it can still remain inapplicable for large environment where n is proportional to the map area.

In this paper, we are proposing to use gradient descent (GD) as an optimization method for the sensor placement problem. At each step of the algorithm, we calculate the analytical derivatives of the coverage function with respect to the position and orientation of each sensor and try to move them in a way that maximizes the overall coverage of the network.

There has been some work conducted on the usage of the classical gradient descent method for the sensor placement problem. Cortes *et al.* [37] proposed a distributed mechanism for maximal coverage in multi-robot sensor systems. They showed that if the coverage performance between sensor and target is based on Euclidean distance, the optimal coverage could be achieved by moving each omni-directional sensor in the direction of its Voronoi cell centroid. In this approach, authors have assumed an omnidirectional coverage area for each sensor, therefore the “dominance region” of each sensor is simulated by its Voronoi cell. This assumption is not valid for directional sensors, which will be considered in our work.

Recently, Schwager *et al.* [38] have proposed another gradient descent method for the unmanned aerial vehicle (UAV) placement problem, but their approach relies on a two dimensional view of the environment, and the covered area of each sensor is strictly binary. As the mentioned approach is designed for placement of the UAVs, each pixel of the camera can cover different area sizes depending on the elevation of the UAV. Therefore, the main notion of coverage in the cost function is the pixel per unit area that the optimization method is trying to minimize. In contrast, the elevation of each camera is fixed in our proposed approach, so the pixel per area value is fixed for all cameras. On the other hand, we consider line-of-sight visibility, such that the optimization method is trying to find the position for which the visibility is maximum.

Unlike previous approaches on sensor placement optimization using the gradient descent method [37,38], we use a realistic model for the terrain and a directional probabilistic sensing model for the coverage of each sensor [8]. We show that in addition to its simplicity, our method can produce results comparable to more sophisticated black box optimization methods, without the need for large computational resources.

The rest of this paper is organized as follows. Section 2 presents our sensor model. In Section 3, the gradient descent method is described in the context of our optimization problem. The presentation of the experimental protocol follows in Section 4. Results are presented in Section 5, where our algorithm is compared with two black box optimization methods, before concluding the paper in Section 6.

## 2. Sensor Model

The proposed sensing model depends on distance, orientation and visibility. Sensors are positioned at a constant height τ above ground level. The sensor position is thus described by a 3D point p = (*x*, *y*, *z*), where (*x*, *y*) are free parameters and *z* = *g*(*x*, *y*) + τ is constrained by the terrain elevation *g*(*x*, *y*) at position (*x*, *y*), as defined by a digital elevation model (DEM). We further assume that the anisotropic properties of sensors are fully defined by a pan angle θ around the vertical axis and a tilt angle *ξ* around the horizontal axis. A sensor network *N* = {**s**_1_, **s**_2_,…, **s***_n_*} of *n* sensors is thus fully specified by 4*n* free parameters **s***_i_* = (**p***_i_*, *θ_i_*, *ξ_i_*), *i* = 1,2,…,*n*, with **p**_i_ = (*x_i_*, *y_i_*).

Now, the coverage *C*(**s***_i_*, **q**) of sensor **s***_i_* at point **q** in the environment can be defined as a function of distance *d*(s*_i_*, q) = ‖**q** − **p***_i_*‖, pan angle *p*(**s***_i_*, **q**) = ∠*_p_* (**q** − **p***_i_*) − *θ_i_*, tilt angle *t*(**s***_i_*, **q**) = ∠*_t_* (**q** − **p***_i_*) − *ξ_i_* and visibility υ(**s***_i_*, **q**) from the sensor:

(1) $$C({\text{s}}_{i},\text{q})=f[\mu_{d}(\|{\text{p}}_{i}-\text{q}\|),\mu_{p}({∠}_{p}(\text{q}-{\text{p}}_{i})-\theta_{i}),\mu_{t}({∠}_{t}(\text{q}-{\text{p}}_{i})-\xi_{i}),\upsilon ({\text{p}}_{i},\text{q})]$$

where ${∠}_{p}(\text{q}-{\text{p}}_{i})=\arctan\left(\frac{y_{\text{q}}-y_{\text{p}i}}{x_{\text{q}}-x_{\text{p}i}}\right)$ is the angle between sensor **s***_i_* and point **q** along the **XY** plane and ${∠}_{t}(\text{q}-{\text{p}}_{i})=\arctan\left(\frac{z_{\text{q}}-z_{\text{p}i}}{\left\|{\text{p}}_{i}-\text{q}\right\|}\right)$ is the angle between sensor **s***_i_* and point **q** along the **XZ** plane. In other words, for **q** to be covered by sensor s*_i_*, we need to take into account its sensing range, sensing angles and visibility. Let *μ_d_*, *μ_p_*, *μ_t_* ∈[0,1] represent some membership functions of the mentioned coverage conditions; then, Equation (1) can be rewritten as a multiplication of these memberships:

$$C({\text{s}}_{i},\text{q})=\mu_{d}\left(\left\|\text{q}-{\text{p}}_{i}\right\|\right)\cdot \mu_{p}({∠}_{p}(\text{q}-{\text{p}}_{i})-\theta_{i})\cdot \mu_{t}({∠}_{t}(\text{q}-{\text{p}}_{i})-\xi_{i})\cdot \upsilon ({\text{p}}_{i},\text{q})$$

The function υ(**p***_i_*, **q**) is binary. Given a sensor position **p***_i_*, if the line-of-sight between sensor **s***_i_* and **q** is obstructed, then we assume that the coverage cannot be met, that is υ(**p***_i_*, **q**) = 0, otherwise the visibility condition is fully respected, that is υ(**p***_i_*, **q**) = 1. In our experiments, we assume that all sensors are one meter above the ground (τ = 1). Memberships *μ_d_*, *μ_p_* and *μ_t_* need to be defined according to their parameters.

At each position **q** of environment *ξ*, the coverage for a single sensor is thus the multiplication of the above four conditions. Value *C* = 1 means full coverage, and *C* = 0 indicates no coverage. Each position **q** is also attributed to another parameter *w_q_* ∈ [0, ∞]. This parameter defines the importance of location **q** for the coverage task. Therefore, higher values of *w_q_* represent higher importance of the location **q** in the goal coverage problem. If more than one sensor covers **q**, then a way to compute the local network coverage *C_l_* is:

(2) $$C_{l}(N,\text{q})=1-\prod_{i=1,...,n}\left(1-C({\text{s}}_{i},\text{q})\right)$$

and the global coverage *C_g_* becomes:

(3) $$C_{g}(N,\Xi )=\frac{1}{\sum_{q\in \Xi }w_{q}}\sum_{q\in \Xi }w_{q}C_{l}(N,\text{q})$$

Given an environment Ξ, the problem statement is thus to determine the sensor network deployment *N* that maximizes global coverage, that is:

(4) $$\underset{N}{\max}C_{g}(N,\Xi )$$

Note that the NP-hardness of the mentioned placement problem could be verified by comparison with the maximum coverage problem, which is known to be NP-hard [39]. In this comparison, each sensor at a specific position and orientation is a sample for a subset that covers a set of locations in the environment, and we want to find the *k* sensor positions that allow a maximum coverage when used together. In this problem, the assumption we are making is more general, as the position and orientation of sensors are continuous and coverage of a sensor for each location is probabilistic.

The membership functions *μ_d_*, *μ_p_* and *μ_t_* can be defined as crisp functions, with a value of 1 when the position is within a fixed sensing range or angle of view and otherwise zero.

(5) $$\mu_{d}\left(\left\|{\text{p}}_{i}-\text{q}\right\|\right)=\{\begin{array}{ll} 1 & \left\|{\text{p}}_{i}-\text{q}\right\|\leq d_{\text{max}}\\ 0 & \;\;\;\;\;\;\;\;\;\text{otherwise} \end{array}$$

(6) $$\mu_{p}({∠}_{p}(\text{q}-{\text{p}}_{i})-\theta_{i})=\{\begin{array}{cc} 1 & \left({∠}_{p}(\text{q}-{\text{p}}_{i})-\theta_{i})\in [-a,a\right]\\ 0 & \text{otherwise} \end{array}$$

(7) $$\mu_{t}({∠}_{t}(\text{q}-{\text{p}}_{i})-\xi_{i})=\{\begin{array}{cc} 1 & \left({∠}_{t}(\text{q}-{\text{p}}_{i})-\xi_{i})\in [-b,b\right]\\ 0 & \text{otherwise} \end{array}$$

**Figure 1.**
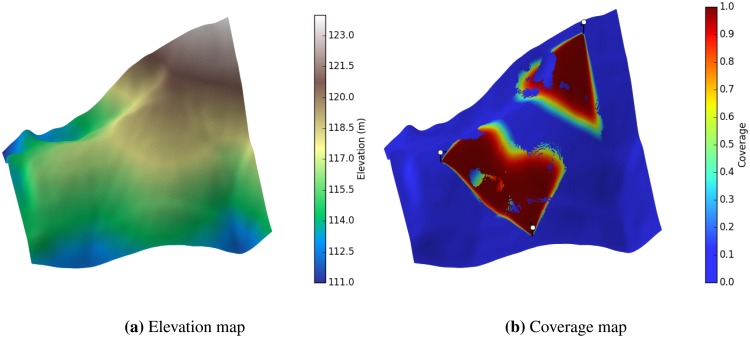
Probabilistic coverage model of a sensor. (**a**) The underlying elevation map used for the experiment; (**b**) Assuming there are three sensors on the map (each shown with a white circle above the ground), the colour shows the different degree of coverage for the whole network. The effect of the visibility function can be seen by the non-visible areas within the coverage region of each sensor.

However, such functions used in a coverage function provide essentially a binary 0/1 signal, which is not a good feedback to use for optimizing functions given its lack of information. Moreover, for a method such as gradient descent, derivable coverage functions are needed. For these reasons, we propose real-valued membership functions that provide a monotonically decreasing membership value over distance and relative angle of position to the sensor (see Figure 1). The value of probabilistic coverage at a given position can be interpreted as the probability of detecting objects of interest from the sensed signal with some given pattern recognition system.

Our proposal is thus to use the following function, based on the well-known sigmoid function, to evaluate the distance membership:

(8) $$\mu_{di}=\mu_{d}(\underset{\phi_{di}}{\underset{︸}{\left\|{\text{p}}_{i}-q\right\|}})=1-\frac{1}{1+\exp(-\beta_{d}(\phi_{di}-\alpha_{d}))}$$

with *α_d_* and *β_d_* being the parameters configuring the membership function. These parameters can be estimated using experimental observations on sensor behaviours (e.g., object recognition rate as a function of distance). Here, parameter *β_d_* controls the slope of the function and *α_d_* determines the distance where the sensor has 50% of its maximum coverage.

As for the pan angle membership functions, we propose another function based on sigmoids:

(9) $$\mu_{pi}=\mu_{p}(\underset{\phi_{pi}}{\underset{︸}{{∠}_{p}(\text{q}-{\text{p}}_{i})-\theta_{i}}})=\frac{1}{1+\exp(-\beta_{p}(\phi_{pi}+\alpha_{p}))}-\frac{1}{1+\exp(-\beta_{p}(\phi_{pi}-\alpha_{p}))}$$

where *α_p_* controls the “width” of the function and *β_p_* controls the slope of the function at the boundaries. Note that the proposed function has the range *φ_pi_* ∈ [−180,180] degrees. Therefore, any calculated angle should be brought into this range accordingly. In the same way, the membership function *μ_t_* is defined as:

(10) $$\mu_{ti}=\mu_{t}(\underset{\phi_{ti}}{\underset{︸}{{∠}_{t}(\text{q}-{\text{p}}_{i})-\xi_{i}}})=\frac{1}{1+\exp(-\beta_{t}(\phi_{ti}+\alpha_{t}))}-\frac{1}{1+\exp(-\beta_{t}(\phi_{ti}-\alpha_{t}))}$$

which has the range *φ_ti_* ∈[−90, 90].

## 3. Gradient Descent Method

Gradient descent (GD) is a classical numerical optimization method used to find the local optimum of an error function. At each step of the algorithm, the gradient of the error function (loss function in our formulation) is calculated and the free parameter of the system is updated to make a small step in the opposite direction to the gradient [40]. Next, the gradient is recalculated for the new solution, and this step is repeated until a maximum number of iterations is reached or the size of the gradient falls beneath a threshold.

### 3.1. Loss Function

Equation (3) defines the objective function for a maximization problem, while the GD method is defined as a minimization method. Therefore, we define the loss function *L*(*N*, *ξ*) as the negative of the coverage function to form a minimization problem. More precisely, the loss function for network *N* and environment Ξ is given by:

(11) $$L(N,\Xi )=\frac{1}{\sum_{\text{q}\in \Xi }w_{\text{q}}}\left[\underset{\text{visible}\;\text{loss}}{\underset{︸}{\sum_{\text{q}\in \Xi }w_{\text{q}}L_{\upsilon }(N,\text{q})}}+\nu \underset{\text{non-visible}\;\text{loss}}{\underset{︸}{\sum_{\text{q}\in \Xi }w_{\text{q}}L_{u}(N,\text{q})}}\right]$$

where *L*_υ_ (*N*, **q**) is the loss incurred by the sensors of network *N* for which point **q** is visible and *L_u_*(*N*, **q**) is the loss incurred by the sensors of network *N* for which point **q** is non-visible. In other words, we take the visibility function away from the formula using two disjoint sets *U_q_* and *V_q_*. Here, *V_q_* contains all of the sensors from which location **q** is visible, and sensors in *U_q_* contain all of the sensors from which location **q** is not visible. More precisely:

(12) $$\begin{array}{c} U_{\text{q}}=\{\text{s}\in N|\upsilon (\text{s},\text{q})=0\}\\ V_{\text{q}}=\{\text{s}\in N|\upsilon (\text{s},\text{q})=1\} \end{array}$$

The visible loss is thus defined as:

(13) $$\begin{array}{ll} L_{\upsilon }(N,\text{q}) & =1-\underset{C_{t}(N,\text{q})}{\underset{︸}{\left[1-\underset{{\text{s}}_{i}\in V_{\text{q}}}{\text{∏}}\underset{L_{\upsilon }({\text{s}}_{i},\text{q}))}{\underset{︸}{\left(1-C({\text{s}}_{i},\text{q})\right)}}\right]}}\\ & =\underset{{\text{s}}_{i}\in V_{\text{q}}}{\text{∏}}[1-\mu_{d}(\|{\text{p}}_{i}-\text{q}\|)\cdot \mu_{p}(\theta_{i}-∠(\text{q}-{\text{p}}_{i}))\cdot \mu_{t}(\xi_{i}-{∠}_{t}(\text{q}-{\text{p}}_{i}))]\\ & =\underset{{\text{s}}_{i}\in V_{\text{q}}}{\text{∏}}[1-\mu_{di}\cdot \mu_{pi}\cdot \mu_{ti}]\\ & =\underset{{\text{s}}_{i}\in V_{\text{q}}}{\text{∏}}L_{\upsilon }({\text{s}}_{i},\text{q}) \end{array}$$

where, for simplicity, we denote *μ_d_* (‖**p***_i_* − **q**‖) as *μ_di_*, *μ_p_*(*θ_i_* − ∠*_p_*(**q** − **p***_i_*)) as *μ_pi_*, and *μ_t_* (*ξ_i_* − ∠*_t_*(**q** − **p***_i_*)) as *μ_ti_*.

The visible loss for the whole network comes to one minus the global coverage defined in Equation (3), which is the coverage function used here as the parameter criterion. However, this measure provides no useful feedback for non-visible positions. Still, these positions are as important as the visible positions in the optimization problem, and their effect should be taken into consideration. In order to provide a better signal to the optimization algorithm, we add a non-visible component to the loss function, which adds the effect of the positions in the environment that are not visible by any sensor or poorly covered by some sensors.

Non-visible loss between sensor **s***_i_* and location **q** is defined as the difference between the current coverage (given by visible loss *L*_υ_ (*N*, **q**)) of point **q** and the coverage that it would have if it was visible from sensor **s***_i_*:

(14) $$\begin{array}{ll} L_{u}({\text{s}}_{i},\text{q}) & =L_{\upsilon }(N,\text{q})-[L_{\upsilon }(N,\text{q})\cdot (1-\mu_{di}\cdot \mu_{pi}\cdot \mu_{ti})]\\ & =\mu_{di}\cdot \mu_{pi}\cdot \mu_{ti}\cdot L_{\upsilon }(N,\text{q}) \end{array}$$

Accordingly, the non-visible loss for a network *N* and location **q** is:

(15) $$L_{u}(N,\text{q})=\sum_{{\text{s}}_{i}\in U_{\text{q}}}L_{u}({\text{s}}_{i},\text{q})=\sum_{{\text{s}}_{i}\in U_{\text{q}}}\mu_{di}\cdot \mu_{pi}\cdot \mu_{ti}\cdot L_{\upsilon }(N,\text{q})$$

### 3.2. Analytical Gradient Descent on Distance, Pan Angle and Tilt Angle

In this section, we calculate the analytical gradient on distance, pan angle and tilt angle for the sensor placement optimization problem.

Given independent variables *x_i_*, *y_i_*, *θ_i_* and *ξ_i_*, we need to obtain partial derivatives of the loss function for all of these variables (see the Appendix for more details). Let:

(16) $${\text{f}}_{i}(N,\text{q})=\prod_{{\text{s}}_{j}\in V_{\text{q}}\backslash \{{\text{s}}_{i}\}}\left[1-\mu_{dj}\cdot \mu_{pj}\cdot \mu_{tj}\right]$$

As there are four free parameters for each sensor, we show the derivative of the overall loss with respect to a generic parameter *ψ* of sensor **s***_i_*. *ψ* can be any of the four parameters (i.e., *x*, *y*, *ψ*;, or *ξ*). The derivations are as follows:

(17) $$\begin{array}{ll} \frac{\partial L(N,\Xi )}{\partial \psi_{i}} & =\frac{1}{\sum_{\text{q}\in \Xi }w_{\text{q}}}\underset{\text{q}\in \Xi }{\text{∑}}w_{\text{q}}\left[\frac{\partial L_{\upsilon }(N,\text{q})}{\partial \psi_{i}}+\nu \frac{\partial L_{u}(N,\text{q})}{\partial \psi_{i}}\right]\\ & =\frac{1}{\sum_{\text{q}\in \Xi }w_{\text{q}}}\underset{\text{q}\in \Xi }{\text{∑}}w_{\text{q}}\left[\frac{\partial [{\text{f}}_{i}\cdot (1-\mu_{di}\cdot \mu_{pi}\cdot \mu_{ti})]}{\partial \psi_{i}}+\nu \frac{\partial \left[\sum_{{\text{s}}_{j}\in U_{\text{q}}}\mu_{dj}\cdot \mu_{pj}\cdot \mu_{tj}\cdot L_{\upsilon }(N,q)\right]}{\partial \psi_{i}}\right]\\ & =\frac{1}{\sum_{\text{q}\in \Xi }w_{\text{q}}}\underset{\text{q}\in \Xi }{\text{∑}}w_{\text{q}}g_{\psi }({\text{s}}_{i},\text{q}) \end{array}$$

where,

(18) $${\text{g}}_{\psi }({\text{s}}_{i},\text{q})=\{\begin{array}{ll} -{\text{f}}_{i}\frac{\partial [\mu_{di}\cdot \mu_{pi}\cdot \mu_{ti}]}{\partial \psi_{i}} & \text{if}\;\upsilon ({\text{s}}_{i},\text{q})=1\\ \nu L_{\upsilon }(N,\text{q})\frac{\partial [\mu_{di}\cdot \mu_{pi}\cdot \mu_{ti}]}{\partial \psi_{i}} & \text{if}\;\upsilon ({\text{s}}_{i},\text{q})=0 \end{array}$$

The derivative of the loss function with respect to parameter ψ can be divided into two parts. The visible loss is constant when the position **q** is not visible from the sensor **s***_i_*, and therefore, the derivative equals zero. Similarly, the non-visible loss has a derivative of zero when sensor **s***_i_* can see position **q**. Therefore, the derivative was divided into two equations using the *g*_ψ_ (**s***_i_*, **q**) function. The ν parameter used in the loss function records the weighting of the non-visible component against the visible loss component.

Therefore, in each step of the GD method, all of the gradients are calculated with respect to the free parameters of each sensor (i.e., *x_i_*, *y_i_*, *θ_i_* and *ξ_i_*), and the position and orientation of all sensors are updated using the pseudocode shown in Algorithm 1.

In this algorithm, $\psi_{i}^{\left(t\right)}$ is a free parameter of sensor *i* at iteration *t* and *η*_ψ_ is the learning rate for the generic parameter *ψ*. Furthermore, *ω* is the momentum parameter used to help the algorithm escape from local minima. The momentum is a classical method used for neural network optimization to encourage faster convergence of the gradient descent algorithm [41]. It increases the step sizes taken for a free parameter if the gradient points in the same direction for several iterations. Therefore, the algorithm can ignore small features in the loss function surface and skip shallow local minima.


**Algorithm 1** The proposed gradient descent method for sensor placement optimization.
  **procedure** GD(*N*, Ξ)   **for**
*t* = 1,…, *max*_*iter*
**do**   **for all s***_i_* ∈ *N*
**do**    
$\Delta \psi_{i}^{\left(t\right)}=\eta_{\psi }\frac{\partial L(N,\xi )}{\partial \psi_{i}}+\omega \Delta \psi_{i}^{\left(t-1\right)},\forall \psi \in \{x,y,\theta ,\xi \}$    
$\psi_{i}^{\left(t+1\right)}=\psi_{i}^{\left(t\right)}-\Delta \psi_{i}^{\left(t\right)},\forall \psi \in \{x,y,\theta ,\xi \}$   **end for**  **end for** **end procedure**


The g_*ψ*_(**s***_i_*, **q**) function needs the value for the derivative of [*μ_di_* · *μ_pi_* · *μ_ti_*] with respect to the free parameters. We have calculated this derivative for all free parameters and reported the result in Equations (21)-(24) (see the Appendix-(24) for more details). In these Equations, dsg(*δ*, *β*, *α*) is an auxiliary function used to simplify the Equations:

(19) $$\text{dsg(}\delta ,\beta ,\alpha )=\frac{\beta \exp(\beta (\delta +\alpha ))}{{\left(1+\exp(\beta (\delta +\alpha ))\right)}^{2}}=\beta \;\text{sig(}\delta ,\beta ,\alpha )[1-\text{sig(}\delta ,\beta ,\alpha )]$$

which corresponds to the derivative of the so-called sigmoid function:

(20) $$\text{sig(}\delta ,\beta ,\alpha )=\frac{1}{1+\exp(\beta (\delta +\alpha ))}$$

To explain the loss function rather informally, the visible loss is the loss incurred by the visible points; similarly the non-visible loss is the loss incurred by the non-visible points. The optimization algorithm tries to maximize the coverage of the visible points through the visible loss and tries to minimize the number of non-visible points through the non-visible loss. Although these two goals seem similar (and they do have similar effects in most of the cases), they are complementary. We will later see in Section 3.3 how they complement each other.

Although we presented the algorithm in a centralized fashion, it is distributed in nature. Each sensor only needs to know its own position, orientation and visible area, in addition to the overlap between its covered area and other sensors in its neighbourhood to calculate its derivatives. Therefore, if we are using mobile sensors capable of communicating with their neighbours (which is essential in most SNs), they can transfer the required information, and each sensor can compute its movement independently.

(21) $$\begin{array}{ll} \frac{\partial [\mu_{di}\cdot \mu_{pi}\cdot \mu_{ti}]}{\partial x_{i}} & =[\text{dsg(}\phi_{di},-\beta_{d},-\alpha_{d})\cdot \frac{\left(x_{i}-x_{q}\right)}{\phi_{di}}\cdot \mu_{pi}\cdot \mu_{ti}\\ & +[\text{dsg(}\phi_{pi},-\beta_{p},-\alpha_{p})-\text{dsg(}\phi_{pi},-\beta_{p},\alpha_{p})]\cdot \frac{y_{\text{q}}-y_{i}}{\phi_{di}^{2}}\cdot \mu_{di}\cdot \mu_{ti}\\ & +[\text{dsg(}\phi_{ti},-\beta_{t},-\alpha_{t})-\text{dsg(}\phi_{ti},-\beta_{t},\alpha_{t})]\cdot \frac{\left(z_{i}-z_{q})(x_{i}-x_{q}\right)}{\phi_{di}[\phi_{di}^{2}+(z_{i}-z_{\text{q}}]}\cdot \mu_{di}\cdot \mu_{pi}] \end{array}$$

(22) $$\begin{array}{ll} \frac{\partial [\mu_{di}\cdot \mu_{pi}\cdot \mu_{ti}]}{\partial y_{i}} & =[\text{dsg(}\phi_{di},-\beta_{d},-\alpha_{d})\cdot \frac{\left(y_{i}-y_{q}\right)}{\phi_{di}}\cdot \mu_{pi}\cdot \mu_{ti}\\ & +[\text{dsg(}\phi_{pi},-\beta_{p},-\alpha_{p})-\text{dsg(}\phi_{pi},-\beta_{p},\alpha_{p})]\cdot \frac{x_{i}-x_{\text{q}}}{\phi_{di}^{2}}\cdot \mu_{di}\cdot \mu_{ti}\\ & +[\text{dsg(}\phi_{ti},-\beta_{t},-\alpha_{t})-\text{dsg(}\phi_{ti},-\beta_{t},\alpha_{t})]\cdot \frac{\left(z_{i}-z_{q})(y_{i}-y_{q}\right)}{\phi_{di}[\phi_{di}^{2}+(z_{i}-z_{\text{q}}]}\cdot \mu_{di}\cdot \mu_{pi}] \end{array}$$

(23) $$\frac{\partial [\mu_{di}\cdot \mu_{pi}\cdot \mu_{ti}]}{\partial \theta_{i}}=[\text{dsg(}\phi_{pi}-\beta_{p},\alpha_{p})-\text{dsg(}\phi_{pi},-\beta_{p},-\alpha_{p})]\cdot \mu_{di}\cdot \mu_{ti},$$

(24) $$\frac{\partial [\mu_{di}\cdot \mu_{pi}\cdot \mu_{ti}]}{\partial \xi_{i}}=[\text{dsg(}\phi_{ti},-\beta_{t},\alpha_{t})-\text{dsg(}\phi_{ti},-\beta_{t},-\alpha_{t})]\cdot \mu_{di}\cdot \mu_{pi}$$

### 3.3. Sanity Checks

In order to check the behaviour of the GD optimization algorithm in real settings, we performed some simple experiments to check if the method performs reasonably in these simple settings, so that we can later extend the experiments to more complex experiments.

**Figure 2.**
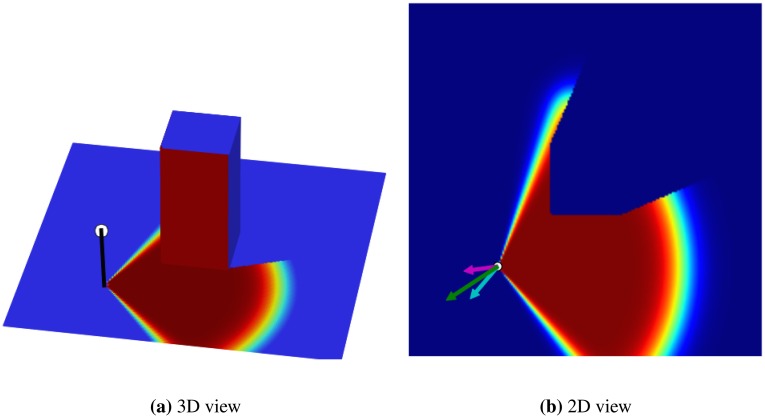
Simple experiment to show the effect of different parts of the loss function, with a 3D view (**a**), and 2D view (**b**) of the covered region of the sensor in a flat environment having a cubic obstacle in the centre. In (b), the cyan arrow shows the non-visible loss gradient, the magenta arrow shows the visible loss gradient and the green arrow is the combined loss gradient aggregating the effect of the two mentioned losses.

**Figure 3.**
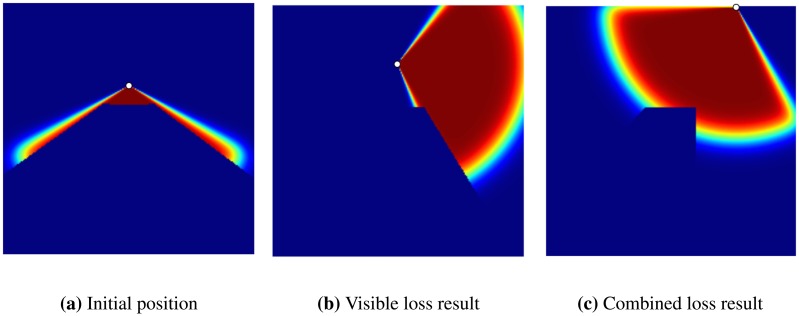
Optimizing with different loss functions: (**a**) the initial position of the sensor over a map (facing one of the sides of the obstacle); (**b**) the final result of the optimization using only the visible loss with a final coverage of 23.29%; (**c**) the final result of the optimization performed using both the visible and non-visible loss parts with a final coverage of 27.47%.

In the first experiment, we examine the effectiveness of the non-visible loss on the overall coverage achieved by the GD algorithm. The setting contains one sensor in a map that has a cube-shaped obstacle in the middle (see Figure 2). Two sets of experiments were performed on the map to optimize the location of the sensors. In the first experiment, we use only the visible loss part of the loss function (Equation (11)). In the second experiment, the combination of both visible and non-visible losses were used for optimization (see Figure 3). In these experiments, the final coverage percentage achieved by the visible and combined loss were 23.29% and 27.47%, respectively. Therefore, we observe that even in the simple example shown, the inclusion of the non-visible loss (in the overall loss function) helps in producing better final results.

**Figure 4.**
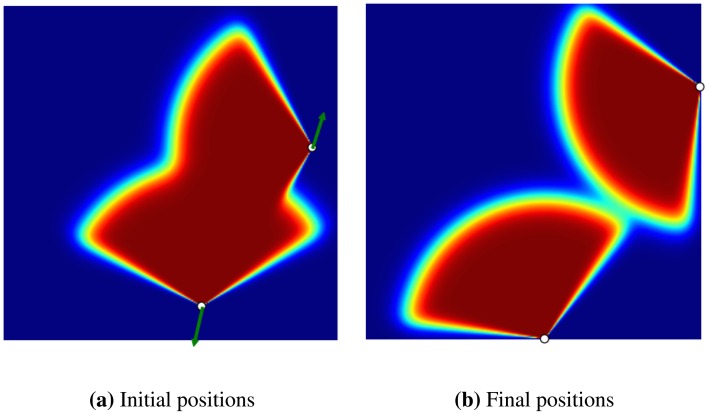
An experiment to show the effect of overlap on coverage over the gradient; (**a**) the initial position of the sensors and the green arrow shows the gradient of the loss function with respect to each sensor; (**b**) the final position of the sensors after the optimization.

In the second experiment, we evaluated the effect of overlap between the coverages of two sensors over the gradient calculated for each one (see Figure 4). In this experiment, two sensors are initially placed in a flat environment (no obstacle), where there is an overlap between the coverage of the two sensors. The gradient calculated for each sensor makes the sensors move away from each other to reach a final position where the total coverage is maximum.

**Figure 5.**
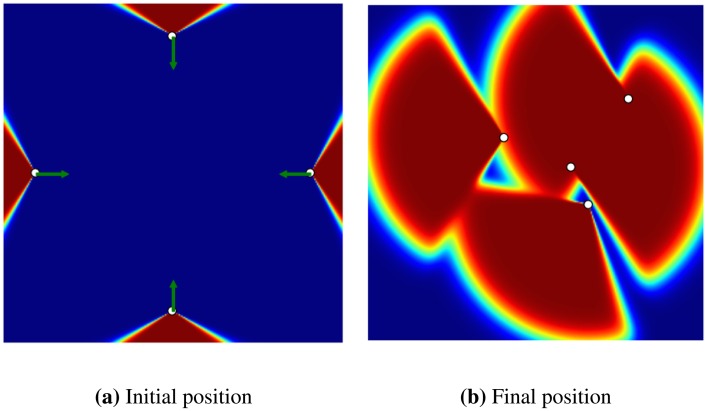
An experiment to show the effect of a coverage gap over the gradient; (**a**) initial positions of the sensors, with the green arrow showing the gradient with respect to each sensor; (**b**) final position of the sensors after the optimization has been completed. As can be seen the initial coverage gap in the centre has been filled by the sensors.

In the final experiment, we show how a coverage gap can be filled with the GD method (see Figure 5). In this experiment, there are four sensors in a flat environment, each heading outward, so creating a coverage gap in the centre. The goal is to determine whether the GD method can detect this gap and displace the sensors in order to cover it. As shown in Figure 5b, the sensors have moved to cover most of the gap.

## 4. Experiments

In order to evaluate the performance of the proposed method, we compared it with two other optimization methods that we already applied to the sensor placement problem, namely simulated annealing (SA) and covariance matrix adaptation evolution strategy (CMA-ES), which we briefly summarize next. CMA-ES was chosen among different population based stochastic optimization methods, as it was shown several times that it has overall superior performance compared to other optimization methods on a variety of standard continuous black-box optimization benchmarks (as an example in [42]). SA was also chosen as it is a classical stochastic optimization method, widely used for global optimization problems. Interested readers are referred to Akbarzadeh *et al.* [7] for more detailed explanations on how each method was used for sensor placement optimization.

### 4.1. Simulated Annealing (SA)

SA [43] is a classical meta-heuristic global optimization method inspired from the annealing process of material in metallurgy. In reality, temperature is the controlling mechanism used to convert material from a high energy state into a low energy, solid condition. This process is imitated in SA, where the temperature controls the number and spread of accessible solutions from a given solution in the search space. SA begins with a high initial temperature to allow a random walk in the search space. As the temperature gradually decreases the system becomes more focused, only allowing moves in the search space which improve the performance of the solution. The process terminates when a temperature close to zero is reached.

SA uses three parameters:

- *M* : the maximum number of iterations for the algorithm.
- *σ_sa_*: the size of the neighbourhood where the subsequent solutions are searched at each iteration of the algorithm.
- *T*(*t*): the temperature function. This function defines the probability of accepting a random move at each iteration of the algorithm.

### 4.2. Covariance Matrix Adaptation Evolution Strategy (CMA-ES)

CMA-ES [44] is an optimization method that belongs to the class of evolutionary computation methods. Like classical quasi-Newton optimization methods, CMA-ES attempts to estimate a second order model of the objective function in an iterative procedure. In contrast to quasi-Newton methods, CMA-ES does not need the gradient of the objective function [45].

The algorithm's parameters include the number of parents (*μ*), the number of offspring (*λ*), the mutation factor (*σ*) and the number of generations through which the algorithm runs. At each generation of the algorithm, a collection of the best *μ* candidate solutions are selected from the set of *λ* offspring of the previous generation. These solutions are then used to update the distribution parameters, which will eventually generate the offspring for the next generation.

### 4.3. Maps

To conduct our experiments, we first selected a mountainous area in North Carolina, USA. The data was provided by a raster layer map in the “OSGeo Edu” dataset (Available at http://grass.osgeo.org/download/sample-data/). More specifically, we focused on a portion of the map that covers a small watershed in a rural area near Raleigh, the capital city of North Carolina. The coordinate system of the map is the NC State Plane (Lambert Conformal Conic projection), metric units and North American Datum (NAD83) geodetic datum. We used four portions of the map for our experiments. The information concerning different selected portions of the map is presented in Table 1. Testing the optimization methods with different map sizes, allows the scalability of each method to be verified.

**Figure 6.**
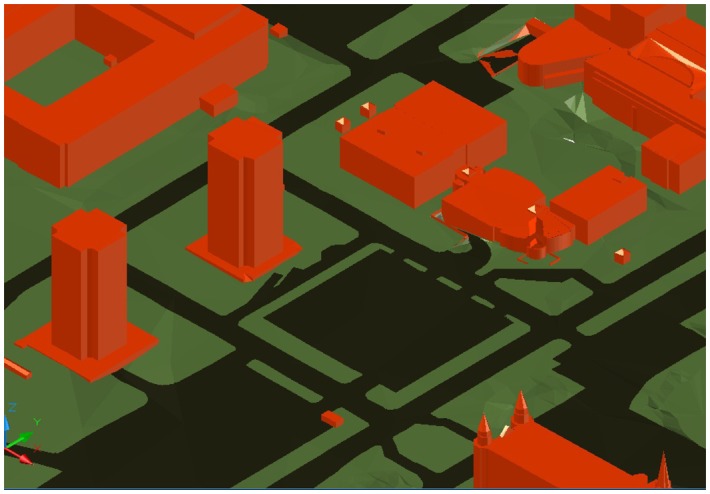
Part of the Université Laval (UL) map, chosen for the weighted experiments. Here, different parts of the map have different weights. Buildings are shown in red and have a weight of *w_q_* = 0.1, the ground is represented in green and has a weight of *w***_q_** = 0.8 and the streets are shown in black and have a weight of *w***_q_** = 0.4.

We also tested the optimization algorithms over a map of Université Laval campus, in Quebec City, Canada. The map of the area is shown in Figure 6. In this experiment, which is an example of a surveillance system for the campus, the goal is two-fold. First, we want to test the performance of different methods in the presence of man-made obstacles *(i.e.*, buildings). Second, the target area is weighted, meaning that each pixel is attributed with a different weight (*w***_q_**) as described in Section 2. For this experiment, we assume that the top of the buildings have low importance in the total coverage (*w***_q_** = 0.1), the streets have an average importance (*w***_q_** = 0.4) and the ground level where the pedestrians walk have the highest importance (*w***_q_** = 0.8). The specifications for the coordinates of the campus maps are also provided in Table 1.

### 4.4. Settings

Sensors are modelled following a description given in Section 2. For a reasonable model of a sensor, we propose to use the parameters shown in Table 2. With these values, the sensors have 50% of the maximum coverage at 30 m or at a sensing angle of 120°.

For SA, the perturbations for positions and orientations follow a Gaussian distribution with standard deviation *σ_sa_*. The optimal value for *σ_sa_* has been established by trial and error (over the range *σ_sa_* ϵ {0.001,0.005,0.01, 0.05, 0.1, 0.2}), and set to *σ_sa_* = 0.01 for each map. CMA-ES is run following recommendations of its author [45], with a population of *λ* = [4 + 3log(N)] offspring and $\mu =\left[\frac{\lambda }{2}\right]$ parents. Here, *N* is the dimensionality of the given problem, determined by the number of sensors in each map. A mutation factor *σ_cma_* = 0.167 is also used. For the GD method, the list of parameters include the learning rates *η_x_* and *η_y_* for positions, the learning rate *η*_*θ*_ for pan angles, the learning rate *η_ξ_* for tilt angles and the weighting parameter *υ*. The values of these parameters used in the experiments have been found by trial and error, with the same values used over all maps. For that, we performed a grid search for all of the parameters over the NC-A map. More precisely, we had a grid search over parameters *η* ∈ {0.001, 0.005, 0.01, 0.05, 0.1, 0.5}, *υ* ∈ {0, 0.5,1,1.5, 2} and *ω* ∈ {0,0.5,1}. Then, we chose the best five selections of the parameters and among them chose the one that performed best (on average) on all of the other maps. All parameters used for the experiments are summarized in Table 3.

The most computationally demanding part of each algorithm lies in calculating the overall coverage for a candidate solution. Therefore, to allow fair comparison between different optimization methods regarding computation requirements, we put a limit on the number of candidate network coverage calculations each method can do. SA and GD methods perform one coverage calculation per iteration; therefore, the maximum number of iterations for these methods equal the maximum number of coverage calculations for each map. The CMA-ES method calculates coverage for each of its offspring at each generation, so this algorithm proceeds for $\frac{t_{\max}}{\lambda }$ iterations on each map, where *t_max_* is the maximum number of iterations and A is the number of offspring. We have reported the maximum iteration for each map and algorithm in Table 4. The maximum number of iterations define the stop criterion for each algorithm on each map.

In the GD method, we added another criteria for the termination of the algorithm. In this algorithm, if the fitness of the candidate solution is not improved after a specific number of iterations, the algorithm will stop and report the best solution found so far. In our implementation, this number of iterations is set to be 50.

For the CMA-ES and SA methods, each sensor placement optimization scheme has been run 30 times, from which are estimated the average and the standard deviation of each method. CPU times are also averaged over the 30 runs, in order to compare the resources required by each method to produce a solution. These time values, reported in Table 5, have been evaluated by running the methods on a single Intel i7 core running at 2.8 GHz.

Comparatively, the running time of GD on a specific map is much less than the other two methods. Therefore, we implemented a restart mechanism for our experiments. More precisely, we accomplished this by calculating, on average, how many runs GD is able to perform with restart to be comparable in terms of the number of evaluations calculated. In stochastic optimization, restarting consists in making several runs of the algorithm and using the best solution of these runs as the final result. Joined to a stop criterion that halts the optimization process as soon as it converges, this leads to better results than one single long run. SA and CMA-ES are executed 30 times, so GD is executed for 30 × the average number of runs with restart executed on each map. In Table 5, for the GD method, we have reported the average coverage percentage between the 30 repetitions with restart as “GD with restart” and the average between all runs as “GD single run average”.

Each optimization method begins the optimization process from an initial network setting. We take the random distribution as our initial setting for the optimization methods. Random distribution is the simplest scenario, meaning that all sensors are randomly distributed in the environment.

All optimization programs are implemented in the Python language. The CMA-ES implementation was taken from Distributed Evolutionary Algorithms in Python (DEAP), available at http://deap.gel.ulaval.ca [46]; a Python library for evolutionary algorithms developed at Université Laval.

## 5. Results

**Figure 7.**
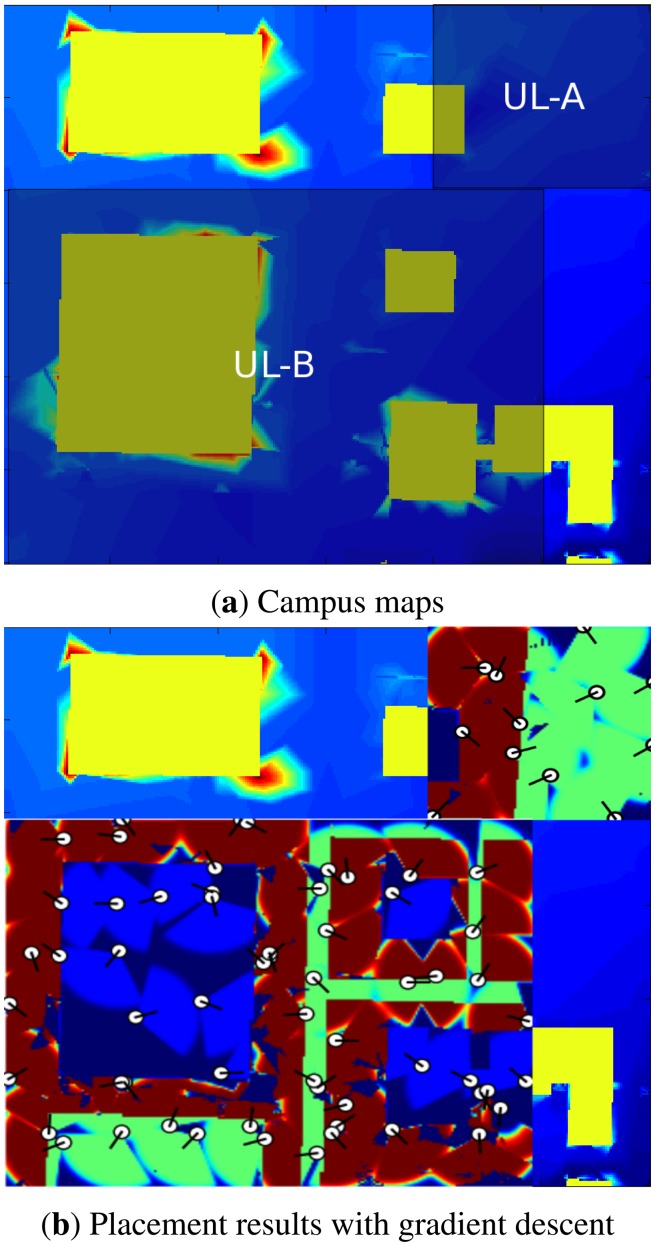
Result of placement on the Université Laval campus map: (**a**) two sub-parts of the maps used for the experiments, the specifications concerning each map are given in Table 1; (**b**) position of the sensors in the best placement obtained by the GD method. Here, white circles represent the position of sensors on the map and the black line connected to each circle shows the direction of each sensor. Different colours present different degrees of coverage using the same colour map as Figure 1.

**Figure 8.**
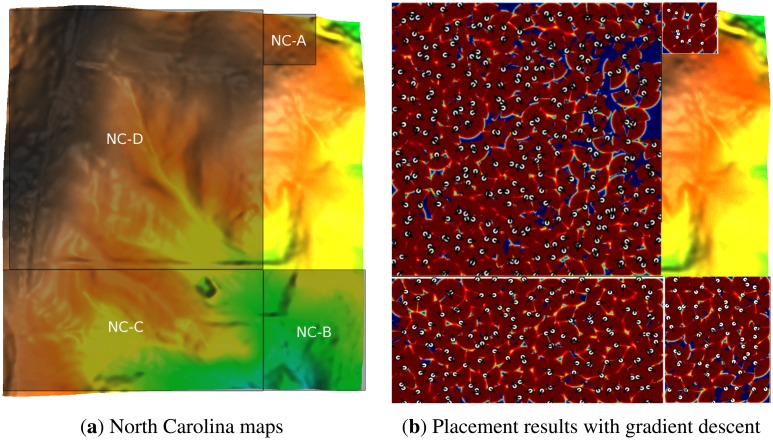
Result of placement on the North Carolina rural area map: (**a**) four sub-parts of the maps used for the experiments (the specification concerning each map is given in Table 1); (**b**) position of the sensors in the best placement obtained by the GD method.

Here, we compare the performance of optimization methods on the mentioned test maps. Each optimization method was run 30 times, except GD, which was run for 30× the average number of runs with restart, from which the average coverage percentage and standard deviation were recorded. A good optimization method should have high coverage and low standard deviation. The results are reported in Table 5. In the experiments, each scheme has been run 30 times, with coverage averages and the corresponding standard deviations reported. Note that 100% coverage is not possible with a finite number of sensors, given its probabilistic nature. Figures 7 and 8 also present the best results obtained by the GD method for each map. There are several aspects that should be pointed out in Figure 7b. First, the coverage of each sensor is scaled by the weight of the area that the sensor is placed on (the same way that the coverage of sensors are weighted in Equation (3)). This gives us the ability to differentiate between areas with different weights. For example, on the top of the buildings (where the weight is smallest), the sensors have blue coverage, or on the roads, the coverage of sensors is green. Next, notice that the area which has lower weights (e.g., the top of the buildings) is not covered as well as the areas with higher weights (e.g., the ground level). This makes sense as the algorithm has balanced the coverage capacity and put more emphasis on the more important areas.

Results indicate that CMA-ES outperformed other methods on three maps and GD performed better on the other three maps. In general, the performance of the three methods is very similar on smaller maps, but on larger maps, the difference is significant. CMA-ES performed better on smaller maps, while GD with restart performed better on larger maps. Still, the difference between the performance of CMA-ES and GD on smaller maps (∼ 1%) is much less than the difference on larger maps (∼ 30%), except for one small map (UL-B) where the difference is larger (∼ 4%). The larger difference of the performance on UL-B map could be related to the abrupt changes of the elevation and visibility on this map, which itself is caused by the abundance of buildings on this map. These changes make the derivative of the objective function discontinuous in the search space; therefore, it will be harder for the GD method to escape local minima. The main difference occurs on the largest map (NC-D) where CMA-ES is unable to obtain a better estimation than the initial random positions. It is well-known that CMA-ES does not scale well in high dimensionality [47]. The reason is that large maps generate high dimensional search spaces (*i.e.*, hundreds of dimensions), and estimating the covariance matrix from a relatively small sample set is brittle. Therefore, the CMA-ES with full covariance matrix is only usable for problems with a small number of sensors (less than 250).

**Figure 9.**
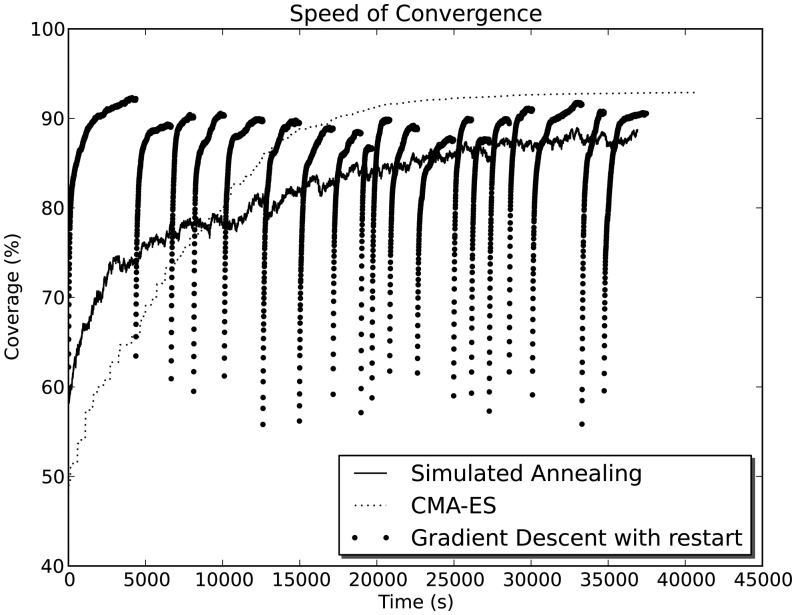
Comparison between speed of convergence for different methods on a sample run for map NC-B.

The other major difference is attributed to the computational demand of different algorithms. SA and CMA-ES consume roughly the same amount of computational power, while GD requires between 13- to 165-times less computation time. For example, Figure 9 compares the speed of convergence between different methods on map NC-B.

## 6. Conclusions

This paper presented an analytical gradient descent (GD) algorithm for optimizing sensor placement. The algorithm was implemented with a realistic model for the environment and a probabilistic model for the sensors. Other optimization methods (CMA-ES and SA) were also implemented and compared with the GD method. In comparison, CMA-ES performed slightly better on smaller maps, while GD performed significantly better on larger maps. Another advantage of the GD method lies in its processing time, as on the tested maps, its performance was between 13- to 165-times superior to that of the other two methods. The final advantage of the algorithm is related to its distributed nature. Although, in this paper, we have tested the algorithm in a centralized fashion, GD has the capability to be executed in a distributed fashion, where each sensor only needs to know the position and coverage of neighbour sensors.

Future work could involve analytically calculating the second order derivative of the objective function to use other gradient-based optimization methods (e.g., Newton's or the quasi-Newton method). Another possible future study could focus on the application of the coverage model to obtain k-coverage over an environment. Therefore, if one sensor fails for any reason, there would be other sensors which could cover the uncovered area.

## Figures and Tables

**Table 1.** Information concerning the test maps used for the experiments.

Method	Map NC-A	Map NC-B	Map NC-C	Map NC-D	Map UL-A	Map UL-B	
West boundary	638,800	638,800	638,300	638,300	245,815	245,615	
East boundary	638,900	639,000	638,800	638,800	245,915	245,865	
South boundary	220,000	220,500	220,500	220,000	5182,750	5,182,550	
North boundary	220,100	220,750	220,750	220,500	5,182,850	5182,750	
Highest elevation	130.65	127.81	131.6	131.55	100.0	107.3	
Lowest elevation	125.95	109.3	103.7	103.76	85.25	82.85	
No. of Columns	100	200	500	500	100	250	
No. of Rows	100	250	250	500	100	200	
No. of pixels	10,000	50,000	125,000	250,000	10,000	50,000	

**Table 2.** The parameter values for a realistic model of a sensor that has 50% of the maximum coverage at 30 m, a pan angle of 120° or a tilt angle of 60°.

**Parameter**	*α_d_*	*β_d_*	*α_p_*	*β_p_*	*α_t_*	*β_t_*
Value	30	1	60	1	30	1

**Table 3.** The parameter values used for simulated annealing, gradient descent and covariance matrix adaptation evolution strategy (CMA-ES) methods.

	SA	GD						CMA-ES
Parameter	*σ_sa_*	*η_x_*	*η_y_*	*η_θ_*	*η_ξ_*	*υ*	*ω*	*σ_cma_*
Value	0.01	0.05	0.05	0.5	0.005	1	0.5	0.167

**Table 4.** Maximum number of iterations for each method on each map.

**Method**	**Map NC-A**	**Map NC-B**	**Map NC-C**	**Map NC-D**	**Map UL-A**	**Map UL-B**
Simulated Annealing (SA)	6000	30,000	103,500	144,000	6000	30,000
Gradient Descent (GD)	6000	30,000	103,500	144,000	6000	30,000
CMA-ES	400	1500	4500	6000	400	1500

**Table 5.** Coverage percentage on the target areas with various numbers of sensors. Results in bold denote the best results that are statistically significant according to the Wilcoxon-Mann-Whitney test (pairwise compared with the other results with *p*-value of 0.05). As the range of the time requirements for different algorithms is large, time is reported either in seconds (s), minutes (m), hours (h) or days (d).

**Method**	**NC-A**	**NC-B**	**NC-C**	**NC-D**	**UL-A**	**UL-B**	
Number of sensors	12	60	150	300	12	60	
Search dimension	48	240	600	1200	48	240	
SA average	88.42%	89.38%	88.98%	84.9%	89.09%	83.56%	
SA SD	1.56%	0.58%	0.43%	0.53%	1.69%	2.47%	
SA CPU time	17.7 m	10.2h	4.1 d	11.1 d	15.1 m	10.1 h	
CMA-ES average	**90.22**%	**92.4**%	91.75%	60.32 %	90.44%	**87.63**%	
CMA-ES SD	1.89%	0.73%	0.65 %	2.3 %	1.47%	1.03%	
CMA-ES CPU time	18.0m	11.0 h	4.4 d	11.4d	15.9m	11.1 h	
GD with restart	88.60%	92.1%	**95.1**%	**91.7**%	**91.38** %	83.17%	
GD single run average	84.99%	90.7%	94.29%	90.3%	86.66%	76.25%	
GD single run SD	0.90%	0.23%	0.12%	0.26%	1.05%	1.64%	
GD average # of restart	13	19	112	165	15	27	
GD CPU time single run	85 s	33.9 m	52.0 m	1.5 h	72 s	22.2 m	

## A. Appendix: Calculation of Derivatives

This section presents the calculation of the analytical derivative of the membership functions (*μ_di_*, *μ_pi_* and *μ_ti_*) with respect to the four free parameters *x_i_*, *y_i_*, *θ_i_* and *ξ_i_* The following function is introduced to simplify the math formulations:

(25) $$\text{dsg(}\delta ,\beta ,\alpha )=\frac{\beta \exp(\beta (\delta +\alpha ))}{{\left(1+\exp(\beta (\delta +\alpha ))\right)}^{2}}=\beta \text{sig(}\delta ,\beta ,\alpha )[1-\text{sig(}\delta ,\beta ,\alpha )]$$

which corresponds to the derivative of the so-called sigmoid function:

(26) $$\text{sig(}\delta ,\beta ,\alpha )=\frac{1}{1+\exp(\beta (\delta +\alpha ))}$$

First, let us develop the derivatives of membership functions with respect to *x_i_* and *y_i_*, that is:

(27) $$\frac{\partial [\mu_{di}\cdot \mu_{pi}\cdot \mu_{ti}]}{\partial x_{i}}=\left[\frac{\partial \mu_{di}}{\partial x_{i}}\cdot \mu_{pi}\cdot \mu_{ti}+\frac{\partial \mu_{pi}}{\partial x_{i}}\cdot \mu_{di}\cdot \mu_{ti}+\frac{\partial \mu_{ti}}{\partial x_{i}}\cdot \mu_{di}\cdot \mu_{pi}\right]$$

(28) $$\frac{\partial [\mu_{di}\cdot \mu_{pi}\cdot \mu_{ti}]}{\partial y_{i}}=\left[\frac{\partial \mu_{di}}{\partial y_{i}}\cdot \mu_{pi}\cdot \mu_{ti}+\frac{\partial \mu_{pi}}{\partial y_{i}}\cdot \mu_{di}\cdot \mu_{ti}+\frac{\partial \mu_{ti}}{\partial y_{i}}\cdot \mu_{di}\cdot \mu_{pi}\right]$$

For the term $\frac{\partial \mu_{di}}{\partial x_{i}}$:

(29) $$\phi_{di}=\|{\text{p}}_{i}-\text{q}\|=\sqrt{{\left(x_{i}-x_{q}\right)}^{2}+{\left(y_{i}-y_{q}\right)}^{2}},$$

(30) $$\frac{\partial \mu_{di}}{\partial \phi_{di}}=\frac{-\beta_{d}\exp(-\beta_{d}(\phi_{di}-\alpha_{d}))}{{\left(1+\exp(-\beta_{d}(\phi_{di}-\alpha_{d}))\right)}^{2}}=\text{dsg(}\phi_{di},-\beta_{d},-\alpha_{d})$$

To obtain the terms $\frac{\partial \mu_{di}}{\partial x_{i}}$ and $\frac{\partial \mu_{di}}{\partial y_{i}}$, we can simply use the chain rule by multiplying the term $\frac{\partial \mu_{di}}{\partial \phi_{di}}$ with additional terms $\frac{\partial \phi_{di}}{\partial x_{i}}$ and $\frac{\partial \phi_{di}}{\partial y_{i}}$, respectively:

(31) $$\frac{\partial \phi_{di}}{\partial x_{i}}=\frac{\partial \sqrt{{\left(x_{i}-x_{q}\right)}^{2}+{\left(y_{i}-y_{q}\right)}^{2}}}{\partial x_{i}}=\frac{\left(x_{i}-x_{q}\right)}{\sqrt{{\left(x_{i}-x_{q}\right)}^{2}+{\left(y_{i}-y_{q}\right)}^{2}}}=\frac{\left(x_{i}-x_{q}\right)}{\phi_{di}}$$

(32) $$\frac{\partial \phi_{di}}{\partial y_{i}}=\frac{\partial \sqrt{{\left(x_{i}-x_{q}\right)}^{2}+{\left(y_{i}-y_{q}\right)}^{2}}}{\partial y_{i}}=\frac{\left(y_{i}-y_{q}\right)}{\sqrt{{\left(x_{i}-x_{q}\right)}^{2}+{\left(y_{i}-y_{q}\right)}^{2}}}=\frac{\left(y_{i}-y_{q}\right)}{\phi_{di}}$$

Consequently, the derivatives of the membership function *μ_di_* can be calculated as:

(33) $$\frac{\partial \mu_{di}}{\partial x_{i}}=\frac{\partial \mu_{di}}{\partial \phi_{di}}\cdot \frac{\partial \phi_{di}}{\partial x_{i}}=\text{dsg(}\phi_{di},-\beta_{d},-\alpha_{d})\cdot \frac{\left(x_{i}-x_{q}\right)}{\phi_{di}}$$

(34) $$\frac{\partial \mu_{di}}{\partial y_{i}}=\frac{\partial \mu_{di}}{\partial \phi_{di}}\cdot \frac{\partial \phi_{di}}{\partial y_{i}}=\text{dsg(}\phi_{di},-\beta_{d},-\alpha_{d})\cdot \frac{\left(y_{i}-y_{q}\right)}{\phi_{di}}$$

For the term $\frac{\partial \mu_{pi}}{\partial x_{i}}$, we first notice that *μ_pi_* = *μ_p_* (*φ_pi_*) where *φ_pi_* = ∠*_p_* (**q**−**p***_i_*) − *θ_i_*. Therefore, we have:

(35) $$\frac{\partial \mu_{pi}}{\partial x_{i}}=\frac{\partial \mu_{pi}}{\partial \phi_{pi}}\cdot \frac{\partial \phi_{pi}}{\partial x_{i}}$$

In the term $\frac{\partial \phi_{pi}}{\partial x_{i}}$ only the pan angle ∠*_p_* (**q** − **p***_i_*) is a function of *x_i_* and *y_i_*:

(36) $${∠}_{p}(\text{q}-{\text{p}}_{i})=\arctan\left(\frac{y_{\text{q}}-y_{i}}{x_{\text{q}}-x_{i}}\right)$$

and, therefore, we have $\frac{\partial \phi_{pi}}{\partial x_{i}}=\frac{\partial ∠(\text{q}-{\text{p}}_{i})}{\partial x_{i}}$. The derivative of ∠*_p_* (**q** − **p***_i_*) with respect to *x_i_* is thus:

(37) $$\frac{\partial {∠}_{p}(\text{q}-{\text{p}}_{i})}{\partial x_{i}}=\frac{\partial {∠}_{p}(\text{q}-{\text{p}}_{i})}{\partial \left(\frac{y_{\text{q}}-y_{i}}{x_{\text{q}}-x_{i}}\right)}\cdot \frac{\partial \left(\frac{y_{\text{q}}-y_{i}}{x_{\text{q}}-x_{i}}\right)}{\partial x_{i}}=\frac{1}{{\left(\frac{y_{\text{q}}-y_{i}}{x_{\text{q}}-x_{i}}\right)}^{2}+1}\cdot \frac{\left(y_{\text{q}}-y_{i}\right)}{{\left(x_{\text{q}}-x_{i}\right)}^{2}}=\frac{y_{\text{q}}-y_{i}}{\phi_{di}^{2}}$$

Similarly, the derivative of ∠*_p_* (**q** − **p***_i_*) with respect to *y_i_* can be obtained:

(38) $$\frac{\partial {∠}_{p}(\text{q}-{\text{p}}_{i})}{\partial y_{i}}=\frac{\partial {∠}_{p}(\text{q}-{\text{p}}_{i})}{\partial \left(\frac{y_{\text{q}}-y_{i}}{x_{\text{q}}-x_{i}}\right)}\cdot \frac{\partial \left(\frac{y_{\text{q}}-y_{i}}{x_{\text{q}}-x_{i}}\right)}{\partial y_{i}}=-\frac{1}{{\left(\frac{y_{\text{q}}-y_{i}}{x_{\text{q}}-x_{i}}\right)}^{2}+1}\cdot \frac{1}{\left(x_{\text{q}}-x_{i}\right)}=-\frac{x_{\text{q}}-x_{i}}{\phi_{di}^{2}}$$

Consequently, we can also develop the terms $\frac{\partial \mu_{pi}}{\partial x_{i}}$ and $\frac{\partial \mu_{pi}}{\partial y_{i}}$ as in the following:

(39) $$\begin{array}{ll} \frac{\partial \mu_{pi}}{\partial x_{i}}=\frac{\partial \mu_{pi}}{\partial \phi_{pi}}\cdot \frac{\partial \phi_{pi}}{\partial x_{i}} & =\frac{\partial \left[\frac{1}{1+\exp(-\beta_{p}(\phi_{pi}+\alpha_{p}))}-\frac{1}{1+\exp(-\beta_{p}(\phi_{pi}-\alpha_{p}))}\right]}{\partial \phi_{pi}}.\frac{\partial {∠}_{p}(\text{q}-{\text{p}}_{i})}{\partial x_{i}}\\ & =-\left[\frac{\beta_{p}\exp(-\beta_{p}(\phi_{pi}+\alpha_{p})))}{{\left(1+\exp(-\beta_{p}(\phi_{pi}+\alpha_{p}))\right)}^{2}}-\frac{\beta_{p}\exp(-\beta_{p}(\phi_{pi}-\alpha_{p})))}{{\left(1+\exp(-\beta_{p}(\phi_{pi}-\alpha_{p}))\right)}^{2}}\right]\cdot \frac{y_{\text{q}}-y_{i}}{\phi_{di}^{2}}\\ & =\left[-\text{dsg}(\phi_{pi},-\beta_{p},-\alpha_{p})+\text{dsg}(\phi_{pi},-\beta_{p,}-\alpha_{p})\right]\cdot \frac{y_{\text{q}}-y_{i}}{\phi_{di}^{2}} \end{array}$$

(40) $$\begin{array}{c} \frac{\partial \mu_{pi}}{\partial y_{i}}=\frac{\partial \mu_{pi}}{\partial \phi_{pi}}\cdot \frac{\partial \phi_{pi}}{\partial y_{i}}=\frac{\partial \left[\frac{1}{1+\exp(-\beta_{p}(\phi_{pi}+\alpha_{p}))}-\frac{1}{1+\exp(-\beta_{p}(\phi_{pi}-\alpha_{p}))}\right]}{\partial \phi_{pi}}.\frac{\partial {∠}_{p}(\text{q}-{\text{p}}_{i})}{\partial y_{i}}\\ =\left[\frac{\beta_{p}\exp(-\beta_{p}(\phi_{pi}+\alpha_{p})))}{{\left(1+\exp(-\beta_{p}(\phi_{pi}+\alpha_{p}))\right)}^{2}}-\frac{\beta_{p}\exp(-\beta_{p}(\phi_{pi}-\alpha_{p})))}{{\left(1+\exp(-\beta_{p}(\phi_{pi}-\alpha_{p}))\right)}^{2}}\right]\cdot \frac{x_{i}-x_{\text{q}}}{\phi_{di}^{2}}\\ =\left[-\text{dsg}(\phi_{pi},-\beta_{p},\alpha_{p})+\text{dsg}(\phi_{pi},-\beta_{p,}-\alpha_{p})\right]\cdot \frac{x_{i}-x_{\text{q}}}{\phi_{di}^{2}} \end{array}$$

For the term $\frac{\partial \mu_{ti}}{\partial x_{i}}$, we have *μ_ti_* = *μ_t_*(*φ_ti_*) where *φ_ti_* = *∠_t_*(**q**−**p***_i_*) − *ξ_i_*:

(41) $$\frac{\partial \mu_{ti}}{\partial x_{i}}=\frac{\partial \mu_{ti}}{\partial \phi_{ti}}\cdot \frac{\partial \phi_{ti}}{\partial x_{i}}$$

In the term $\frac{\partial \phi_{ti}}{\partial x_{i}}$, only the tilt angle ∠*_t_* (**q** − **p**_i_) is a function of *x_i_* and *y_i_* (indirectly through *p_i_*):

(42) $${∠}_{t}(\text{q}-{\text{p}}_{i})=\arctan\left(\frac{z_{\text{q}}-z_{i}}{\left\|{\text{p}}_{i}-\text{q}\right\|}\right)=\arctan\left(\frac{z_{\text{q}}-z_{i}}{\phi_{di}}\right)$$

and, therefore, we have $\frac{\partial \phi_{ti}}{\partial x_{i}}=\frac{\partial {∠}_{t}(\text{q}-{\text{p}}_{i})}{\partial x_{i}}$. If we do the calculation for $\frac{\partial {∠}_{t}(\text{q}-{\text{p}}_{i})}{\partial x_{i}}$, we have:

(43) $$\frac{\partial {∠}_{t}(\text{q}-{\text{p}}_{i})}{\partial x_{i}}=\frac{\partial {∠}_{t}(\text{q}-{\text{p}}_{i})}{\partial \left(\frac{z_{\text{q}}-z_{i}}{\phi_{di}}\right)}\cdot \frac{\partial \left(\frac{z_{\text{q}}-z_{i}}{\phi_{di}}\right)}{\partial \phi_{di}}\cdot \frac{\partial \phi_{di}}{\partial x_{i}}=\frac{1}{1+{\left(\frac{z_{\text{q}}-z_{i}}{\phi_{di}}\right)}^{2}}\cdot \frac{z_{i}-z_{\text{q}}}{\phi_{di}^{2}}\cdot \frac{x_{i}-x_{q}}{\phi_{di}}$$

similarly:

(44) $$\frac{\partial {∠}_{t}(\text{q}-{\text{p}}_{i})}{\partial y_{i}}=\frac{\partial {∠}_{t}(\text{q}-{\text{p}}_{i})}{\partial \left(\frac{z_{\text{q}}-z_{i}}{\phi_{di}}\right)}\cdot \frac{\partial \left(\frac{z_{\text{q}}-z_{i}}{\phi_{di}}\right)}{\partial \phi_{di}}\cdot \frac{\partial \phi_{di}}{\partial y_{i}}=\frac{1}{1+{\left(\frac{z_{\text{q}}-z_{i}}{\phi_{di}}\right)}^{2}}\cdot \frac{z_{i}-z_{\text{q}}}{\phi_{di}^{2}}\cdot \frac{y_{i}-y_{q}}{\phi_{di}}$$

and therefore:

(45) $$\begin{array}{c} \frac{\partial \mu_{ti}}{\partial x_{i}}=\frac{\partial \mu_{ti}}{\partial \phi_{ti}}\cdot \frac{\partial \phi_{ti}}{\partial x_{i}}=\frac{\partial \left[\frac{1}{1+\exp(-\beta_{t}(\phi_{ti}+\alpha_{t}))}-\frac{1}{1+\exp(-\beta_{t}(\phi_{ti}-\alpha_{t}))}\right]}{\partial \phi_{ti}}\cdot \frac{\partial {∠}_{t}(\text{q}-{\text{p}}_{i})}{\partial x_{i}}\\ =-\left[\frac{\beta_{t}\exp(-\beta_{t}(\phi_{ti}+\alpha_{t})))}{{\left(1+\exp(-\beta_{t}(\phi_{ti}+\alpha_{t}))\right)}^{2}}-\frac{\beta_{t}\exp(-\beta_{t}(\phi_{ti}-\alpha_{t})))}{{\left(1+\exp(-\beta_{t}(\phi_{ti}-\alpha_{t}))\right)}^{2}}\right]\cdot \frac{1}{1+{\left(\frac{z_{\text{q}}-z_{i}}{\phi_{di}}\right)}^{2}}\cdot \frac{z_{i}-z_{\text{q}}}{\phi_{di}^{2}}.\frac{x_{i}-x_{q}}{\phi_{di}}\\ =\left[\text{dsg}(\phi_{ti},-\beta_{t},-\alpha_{t})-\text{dsg}(\phi_{ti},-\beta_{t},\alpha_{t})\right]\cdot \frac{\left(z_{i}-z_{\text{q}}\right)\left(x_{i}-x_{\text{q}}\right)}{\phi_{di}\left[\phi_{di}^{2}+{\left(z_{i}-z_{\text{q}}\right)}^{2}\right]} \end{array}$$

also, we have:

(46) $$\begin{array}{c} \frac{\partial \mu_{ti}}{\partial y_{i}}=\frac{\partial \mu_{ti}}{\partial \phi_{ti}}\cdot \frac{\partial \phi_{ti}}{\partial y_{i}}=\frac{\partial \left[\frac{1}{1+\exp(-\beta_{t}(\phi_{ti}+\alpha_{t}))}-\frac{1}{1+\exp(-\beta_{t}(\phi_{ti}-\alpha_{t}))}\right]}{\partial \phi_{ti}}\cdot \frac{\partial {∠}_{t}(\text{q}-{\text{p}}_{i})}{\partial y_{i}}\\ =-\left[\frac{\beta_{p}\exp(-\beta_{t}(\phi_{ti}+\alpha_{t})))}{{\left(1+\exp(-\beta_{t}(\phi_{ti}+\alpha_{t}))\right)}^{2}}-\frac{\beta_{t}\exp(-\beta_{t}(\phi_{ti}-\alpha_{t})))}{{\left(1+\exp(-\beta_{t}(\phi_{ti}-\alpha_{t}))\right)}^{2}}\right]\cdot \frac{1}{1+{\left(\frac{z_{\text{q}}-z_{i}}{\phi_{di}}\right)}^{2}}\cdot \frac{z_{i}-z_{\text{q}}}{\phi_{di}^{2}}.\frac{y_{i}-y_{q}}{\phi_{di}}\\ =\left[\text{dsg}(\phi_{ti},-\beta_{t},-\alpha_{t})-\text{dsg}(\phi_{ti},-\beta_{t},\alpha_{t})\right]\cdot \frac{\left(z_{i}-z_{\text{q}}\right)\left(y_{i}-y_{\text{q}}\right)}{\phi_{di}\left[\phi_{di}^{2}+{\left(z_{i}-z_{\text{q}}\right)}^{2}\right]} \end{array}$$

By substituting Equations (33), (39) and (45) in Equation (27), we obtain the analytical derivative for the *x* position of sensor **s***_i_* to the position **q** in the environment. The derivative to the *y* position can be calculated in the same way, substituting Equations (34), (40) and (46) in Equation (28).

Let us now develop the derivatives of membership functions with respect to *θ_i_*:

(47) $$\frac{\partial \left[\mu_{di}\cdot \mu_{pi}\cdot \mu_{ti}\right]}{\partial \theta_{i}}=\left[\frac{\partial \mu_{di}}{\partial \theta_{i}}\cdot \mu_{pi}\cdot \mu_{ti}+\frac{\partial \mu_{pi}}{\partial \theta_{i}}\cdot \mu_{di}\cdot \mu_{ti}+\frac{\partial \mu_{ti}}{\partial \theta_{i}}\cdot \mu_{di}\cdot \mu_{pi}\right]$$

For the term $\frac{\partial \mu_{di}}{\partial \theta_{i}}$, the change of *θ_i_* will not affect the membership functions *μ_di_* and *μ_ti_*, hence:

(48) $$\frac{\partial \mu_{di}}{\partial \theta_{i}}=\frac{\partial \mu_{ti}}{\partial \theta_{i}}=0$$

For the term $\frac{\partial \mu_{pi}}{\partial \theta_{i}}$:

(49) $$\begin{array}{ll} \frac{\partial \mu_{di}}{\partial \theta_{i}} & =\frac{\partial \mu_{pi}}{\partial \theta_{pi}}\cdot \frac{\partial \phi_{pi}}{\partial \theta_{i}}\\ \frac{\partial \mu_{pi}}{\partial x_{i}} & =\frac{\partial \left[\frac{1}{1+\exp(-\beta_{p}(\phi_{pi}+\alpha_{p}))}-\frac{1}{1+\exp(-\beta_{p}(\phi_{pi}-\alpha_{p}))}\right]}{\partial \phi_{pi}}\cdot (-1)\\ & =-\left[\frac{\beta_{p}\exp(-\beta_{p}(\phi_{pi}+\alpha_{p})))}{{\left(1+\exp(-\beta_{p}(\phi_{pi}+\alpha_{p}))\right)}^{2}}-\frac{\beta_{p}\exp(-\beta_{p}(\phi_{pi}-\alpha_{p})))}{{\left(1+\exp(-\beta_{p}(\phi_{pi}-\alpha_{p}))\right)}^{2}}\right]\\ & =\text{dsg}(\phi_{pi},-\beta_{p},\alpha_{p})-\text{dsg}(\phi_{pi},-\beta_{p,}-\alpha_{p}) \end{array}$$

By substituting Equations (48) and (49) in Equation (47), we obtain the analytical derivative for pan orientation of sensor **s***_i_* to the position **q** in the environment.

Now, the developments of the derivatives of membership functions with respect to *ξ_i_*:

(50) $$\frac{\partial \left[\mu_{di}\cdot \mu_{pi}\cdot \mu_{ti}\right]}{\partial \xi_{i}}=\left[\frac{\partial \mu_{di}}{\partial \xi_{i}}\cdot \mu_{pi}\cdot \mu_{ti}+\frac{\partial \mu_{pi}}{\partial \xi_{i}}\cdot \mu_{di}\cdot \mu_{ti}+\frac{\partial \mu_{ti}}{\partial \xi_{i}}\cdot \mu_{di}\cdot \mu_{pi}\right]$$

For the term $\frac{\partial \mu_{di}}{\partial \xi_{i}}$, the change of *ξ_i_* will not affect the membership functions *μ_di_*, and *μ_pi_*. Hence:

(51) $$\frac{\partial \mu_{di}}{\partial \xi_{i}}=\frac{\partial \mu_{pi}}{\partial \xi_{i}}=0$$

As for the term $\frac{\partial \mu_{ti}}{\partial \xi_{i}}$:

(52) $$\begin{array}{ll} \frac{\partial \mu_{ti}}{\partial \xi_{i}} & =\frac{\partial \mu_{ti}}{\partial \phi_{ti}}\cdot \frac{\partial \phi_{ti}}{\partial \xi_{i}}=\frac{\partial \left[\frac{1}{1+\exp(-\beta_{t}(\phi_{ti}+\alpha_{t}))}-\frac{1}{1+\exp(-\beta_{t}(\phi_{ti}-\alpha_{t}))}\right]}{\partial \phi_{ti}}\cdot (-1)\\ & =-\left[\frac{\beta_{p}\exp(-\beta_{t}(\phi_{ti}+\alpha_{t})))}{{\left(1+\exp(-\beta_{t}(\phi_{ti}+\alpha_{t}))\right)}^{2}}-\frac{\beta_{t}\exp(-\beta_{t}(\phi_{ti}+\alpha_{t})))}{{\left(1+\exp(-\beta_{t}(\phi_{ti}+\alpha_{t}))\right)}^{2}}\right]\\ & =\text{dsg}(\phi_{ti},-\beta_{t},\alpha_{t})-\text{dsg}(\phi_{ti},-\beta_{t,}-\alpha_{t}) \end{array}$$

By substituting Equations (51) and (52) in Equation (50), we obtain the analytical derivative for the tilt orientation of sensor **s**_i_ to the position **q** in the environment.
